# Temporal dynamics unleashed: Elevating variational graph attention

**DOI:** 10.1016/j.knosys.2024.112110

**Published:** 2024-09-05

**Authors:** Soheila Molaei, Ghazaleh Niknam, Ghadeer O. Ghosheh, Vinod Kumar Chauhan, Hadi Zare, Tingting Zhu, Shirui Pan, David A. Clifton

**Affiliations:** a Department of Engineering Science, University of Oxford, United Kingdom; b Department of Data Science and Technology, University of Tehran, Iran; c School of Information and Communication Technology, Griffith University, Australia; d Oxford-Suzhou Centre for Advanced Research, Suzhou, China

**Keywords:** Dynamic graph embedding, Graph variational neural networks, Graph attention network, Deep generative models, Markovian assumptions

## Abstract

This research introduces the Variational Graph Attention Dynamics (VarGATDyn), addressing the complexities of dynamic graph representation learning, where existing models, tailored for static graphs, prove inadequate. VarGATDyn melds attention mechanisms with a Markovian assumption to surpass the challenges of maintaining temporal consistency and the extensive dataset requirements typical of RNN-based frameworks. It harnesses the strengths of the Variational Graph Auto-Encoder (VGAE) framework, Graph Attention Networks (GAT), and Gaussian Mixture Models (GMM) to adeptly navigate the temporal and structural intricacies of dynamic graphs. Through the strategic application of GMMs, the model handles multimodal patterns, thereby rectifying misalignments between prior and estimated posterior distributions. An innovative multiple-learning methodology bolsters the model’s adaptability, leading to an encompassing and effective learning process. Empirical tests underscore VarGATDyn’s dominance in dynamic link prediction across various datasets, highlighting its proficiency in capturing multimodal distributions and temporal dynamics.

## Highlights

• Using GMM in VGAE to tackle multimodality and mitigate prior-posterior misalignment.
• Innovative VarGATDyn model integrates structural and temporal insights.
• Integrating attention mechanisms with Markovian assumptions for dynamic learning.
• VarGATDyn iterates learning at each step for robust representation.
• Achieves top results in dynamic link prediction on seven datasets.

## 1 Introduction

Node representation learning in graph-structured data is crucial across domains like bioinformatics [1], social networks [2], and transportation networks [3]. The goal is to capture a node’s structural properties and feature information in low-dimensional vectors (embeddings) for tasks like node classification [4], [5], [6], link prediction [7], [8], [9], and node clustering [10], [11], [12]. However, most methods focus on static graphs, limiting their utility in dynamic, evolving data scenarios. Dynamic graphs, evolving over time, can be represented by a series of static snapshots. This temporal perspective provides a more realistic portrayal of the complex systems that graphs aim to represent, in contrast to static graphs.

The landscape of dynamic graph representation learning has seen various approaches, each with its set of challenges. Current methodologies can be broadly classified into three categories: temporal smoothness-based methods, Recurrent Neural Network (RNN)-based approaches, and deep generative-based methods. However, each of these approaches presents its own limitations, motivating the exploration of innovative solutions such as our proposed Variational Graph Attention Dynamics (VarGATDyn). Temporal smoothness-based methods aim to enforce the smoothness of node representations from adjacent snapshots [13], [14].

While promising, these methods often falter when faced with significant variations in node evolutionary behaviours [15]. RNN-based approaches, a more common category, store historical snapshots in hidden states [16], [17], [18], [19], [20]. However, RNNs exhibit a data-hungry nature, demanding a large amount of training data. As the number of time steps increases, scalability issues become pronounced, hindering their effectiveness [21]. Deep generative methods, combining temporal smoothness-based and RNN-based approaches with generative models, represent the final category. Despite their potential, these approaches often rely on simplified assumptions and encounter challenges when confronted with complex input data [22], [23]. A notable challenge within this category, particularly in the Variational Graph Autoencoder (VGAE) framework, arises from a misalignment between the prior and estimated posterior distributions within the latent space [24].

VarGATDyn presents a novel approach by integrating attention mechanisms with a Markovian assumption, effectively addressing challenges associated with RNN-based models and overcoming issues related to temporal smoothness in adjacent snapshots within dynamic graphs. The incorporation of the Variational Graph Auto-Encoder (VGAE) framework to capture graph structure at each time step, in conjunction with Graph Attention Networks (GAT) and Gaussian Mixture Models (GMM), establishes a robust foundation for addressing the challenges posed by discrepancies between the prior and estimated posterior distributions in the field of dynamic graph representation learning.

GMMs emerge as a potent solution for effectively modelling multimodality within datasets. The phenomenon of multimodality manifests when a dataset encompasses an overall population and diverse subpopulations, making it challenging to assign each subpopulation to an individual observation. Mixture models, particularly GMMs, present a principled modelling approach tailored to handle such intricate data structures. They serve as universal approximators of densities [25], [26], offering a systematic means to comprehend the complexities inherent in the data. The key contribution of GMM lies in its ability to discern and characterise the underlying probability distribution of observations across the entire population.

In the domain of dynamic graph representation learning, the potency of GMM is pivotal for mitigating challenges linked to the misalignment between prior and estimated posterior distributions. By leveraging GMM, the model gains the capacity to capture the intricate multimodal patterns present in the data. This, in turn, facilitates a more nuanced understanding of the dataset, enabling the model to better approximate the true underlying distribution. As a result, the problem is alleviated, as the model becomes adept at capturing the diverse structural and temporal nuances inherent in dynamic graphs. Our proposed multiple-learning technique further enhances the model’s adaptability by iteratively performing inference, generation, and learning steps, leading to a more thorough and resilient learning process. This comprehensive strategy positions VarGATDyn as a promising solution, outperforming state-of-the-art methods in dynamic link prediction tasks across diverse datasets. The main contributions of this work are:

• Employing GMM within the VGAE framework to learn and effectively model the prior distribution, specifically addressing the multimodal nature of input data and successfully mitigating the problem of misalignment between the prior and estimated posterior distributions.
• Proposing an innovative integrated variational model that captures both structural and temporal properties in dynamic graphs by amalgamating attention mechanisms with Markovian assumptions, enabling the learning of temporal dynamics.
• Iteratively performing inference, generation, and learning steps, repeated m times at each time step, culminating in a comprehensive and resilient learning process, contributing significantly to overcoming the challenges arising from the discrepancy between the prior and estimated posterior distributions.
• Demonstrating the efficacy of our proposed model through its achievement of state-of-the-art performance across multiple dynamic link prediction tasks in seven datasets.

Following this introduction, we delve into related work in Section 2, where we review current approaches and their limitations. In Section 3, we elaborate on our proposed method, VarGATDyn, elucidating its key components and innovations. Subsequently, in Section 4, we offer insights into the experimental details, encompassing datasets, evaluation metrics, and implementation specifics. Finally, in Section 5, we conclude with a summary of our findings and outline directions for future research.

**Fig. 1:**
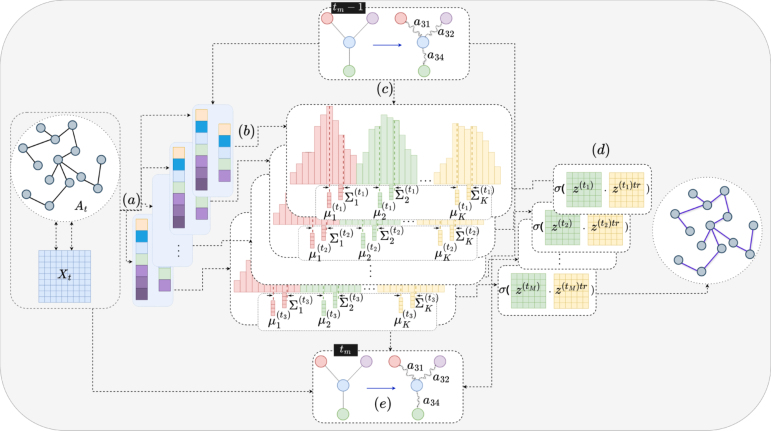
A high-level overview of our method. The figure illustrates the steps of our model in each time step t, utilising the GMM-VGAE structure and the proposed multiple-learning technique. VarGATDyn takes the adjacency matrix $A^{\left(t\right)}$ and feature matrix $X^{\left(t\right)}$ as input (a). After that, it begins the process of replicating the steps of the GMM-VGAE using the multiple-learning technique. Specifically, m replications are performed in order to generate a comprehensive representation of the data. At each of the m replication processes, the input is projected onto a multimodal hidden space with K components (b), which provide a more complex and detailed representation of the data. Each replicate within this structure is conditioned on a variable g, which is derived from the exact replication of the previous time step. This variable is modelled using a GAT framework that incorporates information from the previous time step. This additional latent variable is used in modelling the prior and posterior of GMM-VGAE (c). Afterwards, the adjacency matrix is reconstructed using an inner product decoder for each mth replication process and the learning process is applied (d). Eventually, to generate $g^{\left(t_{m}\right)}$ for the next time step, the adjacency matrix, feature matrix, and the latent variable z of the current replication m, as well as g from the previous exact replication at time step t−1, are used as input to a GAT structure (e). Note that replications are not shown in the structure of producing g for simplicity.

## 2 Related work

In this section, we delve deeper into the landscape of dynamic graph representation learning, highlighting pivotal methodologies and advances in the field. We have organised the discussion around three primary categories: Temporal Smoothness-based Methods, RNN-based Methods (further subdivided into Stacked-RNN-based and Integrated-RNN-based Methods), and Deep Generative-based Methods.

**Temporal smoothness-based methods**: Recently, temporal smoothness-based methods have gained popularity in maintaining representation stability across multiple time steps. Building on the intuition that a network evolves smoothly over time, temporal smoothness-based methods do not build a completely new representation at each time step. For example, Zhou et al. [13] minimise the Euclidean distance between embedding vectors in adjacent time steps and impose a triadic closure concept to preserve the structural evolution of the representation. Another related work is that of Goyal et al. [14], where the authors modify a static Graph Auto-Encoder (GAE) to initialise it with the weights from the previous snapshot conditioning on the fact that significant changes between snapshots are not permitted. On the other hand, Mahdavi et al. [27] propose a method in which the VGAE structure is used to model each graph at each time step, with the assumption that changes in a short period of length l are smooth and continuous. As a result, in order to model the evolution over time, the embeddings produced in each step must be similar to those produced in the l previous steps. Although smooth methods tend to be effective, they often fail when the evolutionary behaviours of nodes change significantly over time.

**Stacked-RNN-based methods**: Recurrent methods learn temporal dynamics by storing historical snapshots in hidden states. The most straightforward way to model discrete dynamic graphs with RNN-based methods is to use a single GNN in each snapshot and then pass the output to an RNN structure for time-series modelling [28]. For example, Seo et al. [19] employ a Graph Convolution Network (GCN) introduced in [29] for structural modelling and a Long Short-Term Memory (LSTM) from [30] for temporal modelling. RgCNN [20] is another RNN-based model that uses a GCN-based approach referred to as PATCHY-SAN for structural modelling and a standard LSTM for temporal modelling. More recent works include that of Manessi et al. [16], where the authors propose two architectures, Waterfall Dynamic-GCN, and Concatenated Dynamic-GCN, that stack a GCN and an LSTM and apply it to each node separately. While these two architectures share many design similarities, the additional skip connection of the GCN in Concatenated Dynamic-GCN distinguishes it from Waterfall Dynamic-GCN.

**Integrated-RNN-based methods**: Using an integrated framework for structural and temporal modelling simultaneously can aid in acquiring a better understanding of modelling dependencies [28]. For example, EvolveGCN [31] combines a GCN and an RNN to update the GCN’s weights with RNN outputs. Another model is GC-LSTM [32] where the authors propose a hybrid of an LSTM and a GCN, in which the graphs of the snapshots are fed into an LSTM. Then a spectral GCN is performed on the LSTM’s hidden layer. Alternatively, LRGCN is another model that combines a relational GCN (R-GCN) for modelling intra- and inter-time relationships with an LSTM for capturing time dependency between graph snapshots [18]. Other works that used GAEs include E-LSTM-D [17], where an LSTM is used in conjunction with an encoder–decoder architecture. In their approach, the encoder is given a series of graphs to map to low-dimensional embeddings. The LSTM then learns network evolution patterns, and the decoder maps the received data to its original space [17]. Despite their high reported performance, existing RNN-based methods entail a large amount of training data and suffer from scalability issues as the number of time steps increases [28].

**Deep Generative-based Methods**: Although previous dynamic graph representation learning methods demonstrate strong performance, they employ deterministic vectors to represent each node in a low-dimensional space. Ignoring uncertainty may cause overfitting, and poor representations [33], [34]. Combining RNN-based or smoothness-based methods with deep generative models can be effective in this field. Deep generative models are known for capturing complex interdependence and interactions between input and output data by considering their distribution [22]. The existing approaches for learning dynamic graph representations using deep generative models typically fall into two categories: those that follow the VGAE framework, and those that use adversarial frameworks. For example, VGRNN [23] is an integrated variational architecture that employs the VGAE framework for structural modelling and an RNN for temporal modelling. Another example is GCN-GAN [35] which is a generative adversarial-based method that employs GCN to investigate the topological properties of each snapshot before employing an LSTM to characterise the evolving properties of dynamic graphs. Generative Adversarial Networks (GAN) are also used in GCN-GAN to enhance the model’s ability to generate subsequent weighted network snapshots. Existing deep generative methods often adopt some oversimplified assumptions that limit their applications to real-world data. For example, most deep generative approaches assume the data comes from simple Gaussian distribution, and do not consider input data intricacy like multimodality [23], [35].

## 3 Proposed method

### 3.1 Notation and problem definition

We define a dynamic graph G as a collection of static graph snapshots, denoted as $G=\left\{G^{\left(1\right)},G^{\left(2\right)},\ldots ,G^{\left(T\right)}\right\}$ for T time steps, where the sets of nodes and edges for time step t are represented by $V^{\left(t\right)}$ and $E^{\left(t\right)}$, respectively, can change over time to account for potential changes in node or edge sets; our proposed model takes a variable-length sequence of adjacency matrices ($A=A^{\left(1\right)},A^{\left(2\right)},\ldots ,A^{\left(T\right)}$) and feature matrices ($X=X^{\left(1\right)},X^{\left(2\right)},\ldots ,X^{\left(T\right)}$) as input, where $A^{\left(t\right)}\in R^{N_{t}\times N_{t}}$ and $X^{\left(t\right)}\in R^{N_{t}\times F}$, with $N_{t}$ representing the number of nodes at time step t, and we assume that the number of features F remains constant over time in this paper. Furthermore, we introduce m={1,…,M} as a variable to indicate the number of replications within a specific time step for the multiple-learning technique, and we denote each iteration within each snapshot as $t_{m}$.

### 3.2 VarGATDyn

VarGATDyn is an integrated method that models the evolution of both topology and node attributes in dynamic graphs using the Markovian assumption. This model utilises a variant of VGAE known as GMM-VGAE [36] to model each snapshot of the graph. The combination of VGAE and GMM is utilised to get the input data’s distribution and attain a fuller understanding of it. To better represent the dynamic structure of the graph over time, GMM-VGAEs are conditioned on an additional latent variable, which is modelled by a GAT framework. Furthermore, to improve the representation of the graph’s dynamic structure, VarGATDyn proposes a technique called multiple-learning which involves iteratively performing inference, generation, and learning processes m times for each time step, resulting in a more robust and comprehensive training process. An overview of the proposed VarGATDyn is shown in Fig. 1.

**Generation.** Our model incorporates three latent variables z, w, and c to seamlessly integrate GMM and VGAE, resulting in a unified GMM-VGAE. The generative process of GMM-VGAE is modelled as follows:

(1) $$p\left(c^{\left(t_{m}\right)};\pi \right)=Cat\left(c^{\left(t_{m}\right)};\pi \right)$$

$$p\left(z^{\left(t_{m}\right)}|c^{\left(t_{m}\right)}\right)=\overset{K}{\underset{k=1}{\prod }}N{\left(z^{\left(t_{m}\right)};\mu_{c_{k}^{\left(t_{m}\right)}},\Sigma_{c_{k}^{\left(t_{m}\right)}}\right)}^{c_{k}^{\left(t_{m}\right)}}$$

Here, Cat is the categorical distribution, $c^{\left(t_{m}\right)}$ is a one-hot vector representing the mixing coefficients of the GMM components at mth iteration of time-step t, π is the mixing probabilities, and K is the number of GMM components. Finally, $\mu_{c_{k}^{\left(t_{m}\right)}}$ and $\Sigma_{c_{k}^{\left(t_{m}\right)}}$ are the mean and diagonal covariance matrix of the kth component, respectively and a neural network (NN) with $w^{\left(t_{m}\right)}$ as input generates them, as shown in Eq. (2).

(2) $$\left\{\mu_{c_{k}^{\left(t_{m}\right)}},\Sigma_{c_{k}^{\left(t_{m}\right)}}\right\}=NN\left(w^{\left(t_{m}\right)}\right);$$

$$w^{\left(t_{m}\right)}∼N\left(0,I\right)$$

In contrast to static GMM-VGAEs, which sample the prior from a standard Gaussian distribution (N(0,I)), our GMM-VGAE uses a new prior sampling process. This process allows the prior distribution’s parameter to be modelled by a function of the previous time step. To achieve this, an additional latent variable, $g^{\left(t_{m}\right)}$, compresses the current step information for the next step. The GAT structure takes multiple inputs, including the adjacency matrix, feature matrix, and the latent variable z from the current time step (t) in the mth iteration, as well as g from the previous time step (t−1) in the exact iteration m. These inputs are used to compute the final representation, as shown in Eq. (3).

(3) $$g^{\left(t_{m}\right)}=GAT\left(A^{\left(t_{m}\right)},X^{\left(t_{m}\right)},z^{\left(t_{m}\right)},g^{\left({\left(t-1\right)}_{m}\right)}\right)$$

In the initial step where the preceding step’s variable g is unavailable, we omit its consideration in our modelling. The construction of the prior distribution can be written as shown in Eq. (4).

(4) $$\left\{\mu_{prior}^{\left(t_{m}\right)},\Sigma_{prior}^{\left(t_{m}\right)}\right\}={Ϝ}_{NN}^{prior}\left(g^{\left({\left(t-1\right)}_{m}\right)}\right)\left\{\pi_{prior}^{\left(t_{m}\right)}\right\}={Ϝ}_{Lin}^{prior}\left(g^{\left({\left(t-1\right)}_{m}\right)}\right)$$

where $\mu_{prior}^{\left(t_{m}\right)}$ and $\Sigma_{prior}^{\left(t_{m}\right)}$ are the mean and covariance of the prior distribution, and $\pi_{prior}^{\left(t_{m}\right)}$ is the mixing probability. The latent variable at the mth iteration of time t is then drawn from a multivariate Gaussian distribution with mean $\mu_{prior}^{\left(t_{m}\right)}$ and covariance $\Sigma_{prior}^{\left(t_{m}\right)}$, i.e., $w^{\left(t_{m}\right)}=N\left(\mu_{prior}^{\left(t_{m}\right)},\Sigma_{prior}^{\left(t_{m}\right)}\right)$. The categorical variable $c^{\left(t_{m}\right)}$ is drawn from a categorical distribution with mixing probability $\pi_{prior}^{\left(t_{m}\right)}$, i.e., $c^{\left(t_{m}\right)}=Cat\left(\pi_{prior}^{\left(t_{m}\right)}\right)$. ${Ϝ}_{NN}^{prior}$ and ${Ϝ}_{Lin}^{prior}$ are functions that produce the parameters of the prior distribution and mixing probability based on the previous time step information.

These functions correspond to a neural network used for generating the parameters of the prior distribution, and a linear function utilised for generating the mixing probability. The prior distribution for the first time step is assumed to be a standard multivariate Gaussian distribution with mean 0 and identity covariance matrix, denoted as N(0,I). If nodes are added at each snapshot, the prior distribution of the added node is also defined as N(0,I). Eliminating a node is equivalent to removing all edges connected to the node, and therefore, the prior probabilities are not affected. The inner-product decoder reconstructs the adjacency matrix between z and its transpose ($z^{tr}$) at each time step, as shown in Eq. (5).

(5) $$p\left(A^{\left(t_{m}\right)}|z^{\left(t_{m}\right)}\right)=\underset{\left(i,j\right)\in E}{\prod }S{\left(z_{i}^{\left(t_{m}\right)tr}z_{j}^{\left(t_{m}\right)}\right)}^{A_{ij}^{\left(t_{m}\right)}}\cdot {\left(1-S\left(z_{i}^{\left(t_{m}\right)tr}z_{j}^{\left(t_{m}\right)}\right)\right)}^{1-A_{ij}^{\left(t_{m}\right)}}$$

In this equation, the symbol S denotes the sigmoid function, whereas the symbol (⋅) stands for the inner product decoder.

**Inference.** The node embedding for dynamic graphs can be calculated by inferring the posterior distribution, which is also a function of g. This architecture design will assist each GMM-VGAE in considering the dynamic graph’s temporal structure. Based on the mean-field variational family,

(6) $$q\left(z^{\left(t_{m}\right)},w^{\left(t_{m}\right)},c^{\left(t_{m}\right)}|A^{\left(t_{m}\right)},X^{\left(t_{m}\right)}\right)=\overset{N}{\underset{i=1}{\prod }}q_{\phi_{z}}\left(z_{i}^{\left(t_{m}\right)}|A_{i}^{\left(t_{m}\right)},X_{i}^{\left(t_{m}\right)}\right)q_{\phi_{w}}\left(w_{i}^{\left(t_{m}\right)}|A_{i}^{\left(t_{m}\right)},X_{i}^{\left(t_{m}\right)}\right)$$

$$p_{\beta }\left(c_{i}^{\left(t_{m}\right)}|z_{i}^{\left(t_{m}\right)},w_{i}^{\left(t_{m}\right)}\right)$$

Here, N is the number of data points, $\phi_{z}$ and $\phi_{w}$ are the variational parameters associated with z and w, respectively. The set of GMM parameters related to each mixture component is denoted as β. The conditional distributions of z|x and w|x are defined as Gaussian distributions, with parameters generated via GNNs using the adjacency matrix $A^{\left(t_{m}\right)}$ and the concatenation of the node feature matrix $X^{\left(t_{m}\right)}$ with the latent variable g from the previous time step. The conditional posterior distribution of c|z,x is given by Eq. (7).

(7) $$p_{\beta }\left(c_{j}^{\left(t_{m}\right)}=1|z^{\left(t_{m}\right)},w^{\left(t_{m}\right)}\right)=\frac{\pi_{j}p\left(z^{\left(t_{m}\right)}|c_{j}^{\left(t_{m}\right)}=1,w^{\left(t_{m}\right)};\beta \right)}{\overset{K}{\underset{k=1}{\sum }}\pi_{k}^{\left(t_{m}\right)}p\left(z^{\left(t_{m}\right)}|c_{k}^{\left(t_{m}\right)}=1,w^{\left(t_{m}\right)};\beta \right)}$$

Here c is a binary variable indicating the assignment of data points to mixture components.

**Learning.** Our proposed model comprises two essential components for effective learning: (1) The incorporation of both structural and temporal aspects of modelling, and (2) The utilisation of multiple-learning techniques to create a comprehensive model of the input data. To incorporate both structural and temporal aspects in our modelling approach, we take two different points of view. From the structural point of view, we calculate the Evidence Lower Bound (ELBO) of the GMM-VGAE for each time step. The ELBO of the GMM-VGAE loss function for each time step is calculated as follows,

(8) $$p\left(A^{\left(t_{m}\right)},z^{\left(t_{m}\right)},w^{\left(t_{m}\right)},c^{\left(t_{m}\right)}\right)=$$

$$p\left(w^{\left(t_{m}\right)}\right)p\left(c^{\left(t_{m}\right)}\right)p\left(z^{\left(t_{m}\right)}|w^{\left(t_{m}\right)},c^{\left(t_{m}\right)}\right)p\left(A^{\left(t_{m}\right)}|z^{\left(t_{m}\right)}\right)$$

**Table 1:** A summary of the datasets used in the experiments in terms of nodes, edges, density, snapshots, and node, where applicable.

Dataset	Number of snapshots	Number of nodes	Number of edges	Number of node attributes
Enron	11	184	115–266	–
Colab	10	315	165–308	–
Facebook	9	663	844–1068	–
LFB	36	45 435	180 011	–
UCI	7	537–1899	59 835	–
Yelp	12	6569	95 361	–
Cora	6	500–2708	406–5429	1433

**Table 2:** AUC and AP scores of link prediction on dynamic graphs. The best results are highlighted.

	Model	Enron	Colab	Facebook	UCI	Cora
AP	DynAE	76.00 ± 2.0	64.02 ± 2.1	56.04 ± 0.9	91.12 ± 0.5	57.11 ± 0.5
	DynRNN	85.61 ± 2.5	78.95 ± 2.7	75.88 ± 0.8	89.21 ± 0.7	80.75 ± 0.7
	DynAERNN	89.37 ± 0.7	81.84 ± 2.1	78.55 ± 1.3	89.92 ± 0.4	82.93 ± 0.6
	DySAT	93.06 ± 0.2	90.40 ± 0.3	80.39 ± 0.3	85.01 ± 0.2	87.73 ± 0.2
	HTGN	94.31 ± 0.2	91.91 ± 0.2	83.80 ± 0.3	86.72 ± 0.1	90.12 ± 0.2
	VGRNN	93.29 ± 0.7	87.77 ± 0.7	89.04 ± 0.8	91.83 ± 0.5	93.32 ± 0.4
	SI-VGRNN	94.44 ± 0.7	88.36 ± 1.2	90.19 ± 0.8	93.16 ± 0.5	96.68 ± 0.4
	AMCNet	93.10 ± 0.7	90.06 ± 7.0	86.02 ± 1.0	92.25 ± 0.8	90.13 ± 1.2

	VarGATDyn	**98.61 ± 0.7**	**97.78 ± 0.8**	**96.12 ± 0.7**	**96.17 ± 0.5**	**99.31 ± 0.4**

AUC	DynAE	74.22 ± 2.8	63.14 ± 2.1	56.06 ± 1.2	91.89 ± 0.8	57.13 ± 0.6
	DynRNN	86.41 ± 2.2	75.7 ± 3.5	73.18 ± 3.1	89.27 ± 1	80.10 ± 0.7
	DynAERNN	87.43 ± 0.9	76.06 ± 2.1	76.02 ± 1.1	90.08 ± 0.6	78.00 ± 0.5
	DySAT	93.06 ± 0.3	87.25 ± 0.2	76.88 ± 0.2	86.73 ± 0.3	85.3 ± 0.1
	HTGN	94.17 ± 0.2	89.26 ± 0.2	83.70 ± 0.3	87.25 ± 0.2	89.73 ± 0.3
	VGRNN	93.10 ± 0.5	85.95 ± 0.6	89.47 ± 0.6	92.01 ± 0.5	94.41 ± 0.5
	SI-VGRNN	93.93 ± 1.0	85.45 ± 1.0	90.94 ± 0.9	93.5 ± 0.5	97.17 ± 0.4
	AMCNet	93.00 ± 0.9	91.22 ± 0.9	87.08 ± 1.1	93.31 ± 0.8	92.05 ± 0.9

	VarGATDyn	**98.71 ± 0.8**	**97.80 ± 0.8**	**96.89 ± 0.7**	**96.99 ± 0.4**	**98.72 ± 0.3**

Based on Eq. (6) and Eq. (8), the lower bound for each snapshot can be written as Eq. (9).

(9) $$L_{ELBO}^{\left(t_{m}\right)}=E_{q}\left[\frac{p\left(A^{\left(t_{m}\right)},z^{\left(t_{m}\right)},w^{\left(t_{m}\right)},c^{\left(t_{m}\right)}\right)}{q\left(z^{\left(t_{m}\right)},w^{\left(t_{m}\right)},c^{\left(t_{m}\right)}|A^{\left(t_{m}\right)}\right)}\right]=$$

$$E_{q}\left[\log p\left(A^{\left(t_{m}\right)}|z^{\left(t_{m}\right)}\right)\right]-E_{q}\left[\log\frac{q\left(z^{\left(t_{m}\right)}|A^{\left(t_{m}\right)},X^{\left(t_{m}\right)}\right)}{p\left(z^{\left(t_{m}\right)}|w^{\left(t_{m}\right)},c^{\left(t_{m}\right)}\right)}\right]-$$

$$E_{q}\left[\frac{q\left(w^{\left(t_{m}\right)}|A^{\left(t_{m}\right)},X^{\left(t_{m}\right)}\right)}{p\left(w^{\left(t_{m}\right)}\right)}\right]-E_{q}\left[\frac{p\left(c^{\left(t_{m}\right)}|z^{\left(t_{m}\right)},w^{\left(t_{m}\right)}\right)}{p\left(c^{\left(t_{m}\right)}\right)}\right]$$

Our ELBO’s first term, which assesses the discrepancy between the input data and their reconstruction, has the exact same structure as the first term of the ELBO’s VGAE. The second term measures the similarity between $q\left(z^{\left(t_{m}\right)}|A^{\left(t_{m}\right)},X^{\left(t_{m}\right)}\right)$ and $p\left(z^{\left(t_{m}\right)}|w^{\left(t_{m}\right)},c^{\left(t_{m}\right)}\right)$. To approximate this term, Monte Carlo can be used as shown in Eq. (10).

(10) $$E_{q}\left[KL\left(q\left(z^{\left(t_{m}\right)}|A^{\left(t_{m}\right)},X^{\left(t_{m}\right)}\right)∥p\left(z^{\left(t_{m}\right)}|w^{\left(t_{m}\right)},c^{\left(t_{m}\right)}\right)\right)\right]\approx \frac{1}{J}\overset{J}{\underset{j=1}{\sum }}\overset{K}{\underset{k=1}{\sum }}p\left(c_{k}^{\left(t_{m}\right)}=1|w_{j}^{\left(t_{m}\right)},z_{j}^{\left(t_{m}\right)}\right)$$

$$KL\left(q\left(z^{\left(t_{m}\right)}|A^{\left(t_{m}\right)},X^{\left(t_{m}\right)}\right)∥p\left(z^{\left(t_{m}\right)}|w_{j}^{\left(t_{m}\right)},c_{k}^{\left(t_{m}\right)}=1\right)\right)$$

The third term measures the similarity between $q\left(w^{\left(t_{m}\right)}|A^{\left(t_{m}\right)},X^{\left(t_{m}\right)}\right)$ and $p\left(w^{\left(t_{m}\right)}\right)$ which is equal to calculating KL-divergence for them as shown in Eq. (11).

(11) $$E_{q}\left[\frac{q\left(w^{\left(t_{m}\right)}|A^{\left(t_{m}\right)},X^{\left(t_{m}\right)}\right)}{p\left(w^{\left(t_{m}\right)}\right)}\right]=KL\left(q\left(w^{\left(t_{m}\right)}|A^{\left(t_{m}\right)},X^{\left(t_{m}\right)}\right)∥p\left(w^{\left(t_{m}\right)}\right)\right)$$

The last term is a KL divergence of categorical distributions as shown in Eq. (12).

(12) $$KL\left(p\left(c^{\left(t_{m}\right)}|z^{\left(t_{m}\right)},w^{\left(t_{m}\right)}\right)∥p\left(c^{\left(t_{m}\right)}\right)\right)=\overset{K}{\underset{i=1}{\sum }}p\left(c_{i}^{\left(t_{m}\right)}=1\right)\log\frac{p\left(c_{i}^{\left(t_{m}\right)}=1\right)}{p\left(c_{i}^{\left(t_{m}\right)}=1|z^{\left(t_{m}\right)},w^{\left(t_{m}\right)}\right)}$$

From a temporal point of view, we adjust the loss function to embody the Markovian assumption. To accomplish this, we add the loss function of the previous time step, referred to as Temporal Loss (TL), to the loss function of the current time step, referred to as Snapshot Loss (SL). The loss function at time t is defined as the sum of SL and TL, as shown in Eq. (13).

(13) $$L^{\left(t_{m}\right)}=\alpha .TL+\left(1-\alpha \right).SL$$

Here the hyperparameter α controls the emphasis on each component. The multiple-learning technique replicates the processes of inference, generation, and learning m times for each time step. In this technique, the loss function in the examined time step is the sum of the loss functions of the m execution times. This multiple-learning technique provides several benefits. First, by iteratively performing the inference, generation, and learning steps, the model can better capture complex temporal dependencies and fluctuations in the data. Second, it provides a more comprehensive and robust training process, reducing the risk of overfitting and improving generalisation performance. Third, it allows for the exploitation of multiple paths of exploration, which can improve the diversity of generated samples and lead to better representation learning. The *SL* at each time step is defined as shown in Eq. (14).

(14) $$SL=\overset{M}{\underset{m=1}{\sum }}L_{ELBO_{m}}$$

Here $L_{ELBO_{m}}$ represents the loss function of the mth replication of each snapshot. The total loss function of the model is calculated as the sum of the loss functions of each snapshot. Thus, the total loss function can be written as Eq. (15).

(15) $$L^{\left(total\right)}=\overset{T}{\underset{t=1}{\sum }}\overset{M}{\underset{m=1}{\sum }}L^{\left(t_{m}\right)}$$

**Fig. 2:**
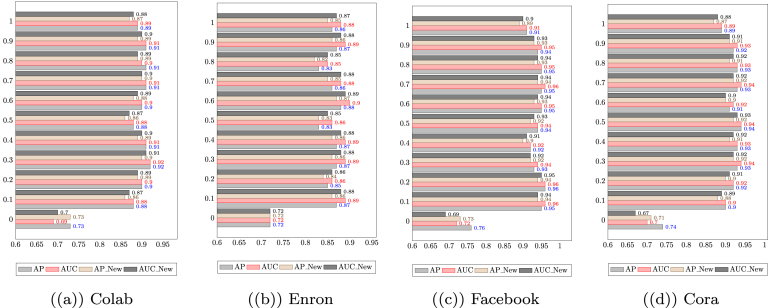
Impact of using various alphas on the two dynamic prediction tasks. AP New and AUC New refer to the metrics for the new link prediction task, while AUC and AP refer to the metrics for the link prediction task. The results are reported in terms of the average of 500 epochs.

**Fig. 3:**
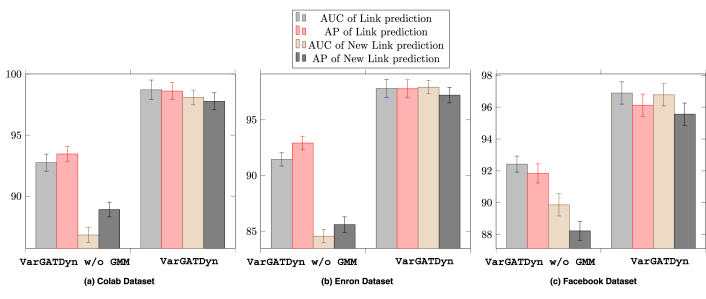
A comparison of VarGATDyn’s performance in two modes, with and without GMM. The colours represent the AP and AUC criteria for both link prediction and new link prediction tasks.

**Table 3:** AUC and AP scores of new link prediction on dynamic graphs.

	Model	Enron	Colab	Facebook	UCI	Cora
AP	DynAE	66.50 ± 1.9	58.82 ± 2.1	54.57 ± 0.9	89.65 ± 0.6	56.65 ± 0.5
	DynRNN	80.96 ± 2.6	75.34 ± 2.6	75.52 ± 0.8	86.86 ± 0.6	80.01 ± 0.8
	DynAERNN	85.16 ± 0.8	77.68 ± 1.9	78.70 ± 1.2	88.15 ± 0.5	82.34 ± 0.6
	DySAT	86.83 ± 0.4	83.47 ± 0.4	78.34 ± 0.5	83.94 ± 0.3	87.15 ± 0.3
	HTGN	90.62 ± 0.4	84.06 ± 0.3	81.70 ± 0.2	84.26 ± 0.3	89.83 ± 0.3
	VGRNN	87.57 ± 0.7	79.63 ± 0.7	86.30 ± 0.9	89.48 ± 0.6	93.21 ± 0.5
	SI-VGRNN	87.88 ± 0.8	81.26 ± 1.2	86.72 ± 0.9	90.07 ± 0.6	95.32 ± 0.6
	AMCNet	92.12 ± 0.8	88.00 ± 1.0	85.00 ± 0.8	92.21 ± 0.7	92.08 ± 0.6

	VarGATDyn	**97.77 ± 0.7**	**97.20 ± 0.7**	**95.56 ± 0.7**	**96.17 ± 0.5**	**98.98 ± 0.5**

AUC	DynAE	66.10 ± 3.1	58.14 ± 2.8	54.62 ± 1.9	89.94 ± 1.2	56.27 ± 0.9
	DynRNN	83.20 ± 2.6	71.71 ± 3.7	73.32 ± 3.2	87.27 ± 1.3	79.94 ± 0.8
	DynAERNN	83.77 ± 1.2	71.99 ± 2.3	76.35 ± 1.3	88.29 ± 0.8	77.36 ± 0.8
	DySAT	87.94 ± 0.5	79.74 ± 0.5	74.97 ± 0.6	84.20 ± 0.4	86.11 ± 0.6
	HTGN	91.26 ± 0.3	81.74 ± 0.3	82.21 ± 0.4	84.98 ± 0.4	87.85 ± 0.4
	VGRNN	88.43 ± 0.7	77.09 ± 0.6	87.20 ± 0.7	89.93 ± 0.5	94.94 ± 0.4
	SI-VGRNN	88.60 ± 0.9	77.95 ± 0.7	87.74 ± 0.6	90.45 ± 0.5	96.36 ± 0.6
	AMCNet	93.01 ± 0.7	89.10 ± 0.9	86.00 ± 0.7	93.00 ± 0.6	93.23 ± 0.5

	VarGATDyn	**98.09 ± 0.6**	**97.91 ± 0.6**	**96.78 ± 0.7**	**96.08 ± 0.6**	**98.99 ± 0.5**

## 4 Experimental details

### 4.1 Datasets

The proposed VarGATDyn is validated and compared to other baselines on communication networks and citation networks. The details of the datasets used are provided in this section (see Table 1).

**Communication networks.**

**UCI**[37]: In UCI, message interaction data from an online community has been collected. This information was collected over a seven-day period. Each day represents a different snapshot in the graph’s evolution. This network begins with 537 nodes and ends with 1899 nodes.

**Enron**[37]: The Enron dataset is derived from emails sent and received by Enron employees. Nodes represent employees, and edges represent emails between employees. Cleaning and constructing the necessary structure for applying the algorithm are carried out by [23], [38], [39].

**Facebook**[40]: Facebook stores information about the posts made on this social media platform. There are 663 nodes and 1068 edges in this dataset. The cleaning and preparation of the data are similar to that described in [38], [39].

**LFB**[40]: This dataset is a larger-scale version of the Facebook dataset containing 45,435 nodes and 180,011 edges. The data cleaning and preparation methods adopted in this version closely follow the procedures outlined in [38], [39]. The dataset includes 36 snapshots capturing activations over the past three years. The LFB dataset features a significant number of users, but the interlinking between them is sparse.

**Yelp**[41]: Yelp, as part of Round 11 in the Yelp Dataset Challenge, functions as a dynamic rating network that diligently collects and records user-generated ratings for various businesses over a defined time period. This unique platform serves as a valuable repository of user experiences and opinions, empowering consumers to make informed decisions and aiding businesses in gaining valuable insights into their performance.

**Citation networks.**

**Colab**[39]: Colab consists of 315 authors and relationships between them. Each node in this dataset represents an author, and each edge depicts a collaboration between two authors. This data set is gathered between 2000 to 2009.

**Cora**[42]: This dataset is basically a static citation network. Cora consists of 2708 nodes representing the publications with a 1433-dimensional binary feature vector. To use Cora dynamically, we preprocess the data in the same way that [23], [43] describes. We added 500 nodes with their associated edges to the dynamic network at each temporal snapshot (208 nodes for the last snapshot), using the node indexes as their arrival order, and six snapshots of the dynamic graph were taken, beginning with 500 nodes and ending with 2708 nodes.

### 4.2 Baselines

We compare VarGATDyn with the following baselines and state-of-the-art graph representation learning methods.

• **dygraph2vec**[44]: This is an auto-encoder framework. We assess our model against three variants of dyngraph2vec: (1) dyngraph2vecAE, utilising fully connected layers, (2) dyngraph2vecRNN, employing an LSTM, and (3) dyngraph2vecAERNN, which is a combination of dyngraph2vecAE and dyngraph2vecRNN.
• **DySAT**[21]: a model that utilises joint self-attention to learn node embedding in the structural and temporal dynamics dimensions.
• **HTGN**[45]: a model that employs a hyperbolic GNN in conjunction with a hyperbolic GRNN for temporal link prediction.
• **(SI-)VGRNN**[23]: a model based on variational auto-encoder architecture integrated into a Graph Recurrent Neural Network (GRNN) structure to model the temporal evolution of the graph. They also develop semi-implicit variational inference in the SI-VGRNN version for further flexibility of modelling.
• **AMCNet**[46]: The approach proposes the Attentional Multi-scale Co-evolving Network (AMC-Net) to address dynamic link prediction by modelling correlations among evolving dynamics across various structural scales. This involves a motif-based graph neural network with multi-scale pooling for structural insights and a hierarchical attention-based sequence-to-sequence model to analyse the evolution dynamics at different scales.

### 4.3 Tasks and evaluation metrics

We evaluate VarGATDyn and compare it to the baseline methods in two link prediction tasks in dynamic graphs. The first task is *dynamic link prediction*, which predicts all links in $G^{\left(T_{M}\right)}$. The second task is referred to as *dynamic new link prediction*, and it aims to predict links in $G^{\left({\left(T+1\right)}_{M}\right)}$ that do not exist in $G^{\left(T_{M}\right)}$. We compare our suggested method with the baselines in link prediction and new link prediction tasks using the Average Precision (AP) and the Area Under the Receiver Operating Characteristic Curve (AUC) metrics from [47]. For dynamic (new) link prediction, all (new) edges are assumed to be true edges, and the same number of non-links are randomly selected to compute AP and AUC scores. In all of our experiments, we test the model on the last three snapshots of dynamic graphs while learning the parameters of the models based on the rest of the snapshots. For datasets without node attributes, we assign the identity matrix with dimensions equal to the number of nodes at each time step as the node attributes.

**Table 4:** Comparison results of AUC and AP for link prediction and new link prediction tasks on large datasets.

	Metric	VarGATDyn	SI-VGRNN	VGRNN	DySAT
LFB	AUC of Link prediction	**86.37**	80.27	79.11	76.88
	AP of Link prediction	**87.04**	82.01	81.40	80.39
	AUC of new Link prediction	**84.47**	77.42	79.61	76.04
	AP of new Link prediction	**87.09**	80.12	76.33	79.35

Yelp	AUC of Link prediction	**82.15**	74.54	72.87	69.91
	AP of Link prediction	**83.42**	75.24	72.64	70.04
	AUC of new Link prediction	**82.03**	74.72	71.99	69.21
	AP of new Link prediction	**83.12**	75.87	72.34	69.05

**Fig. 4:**
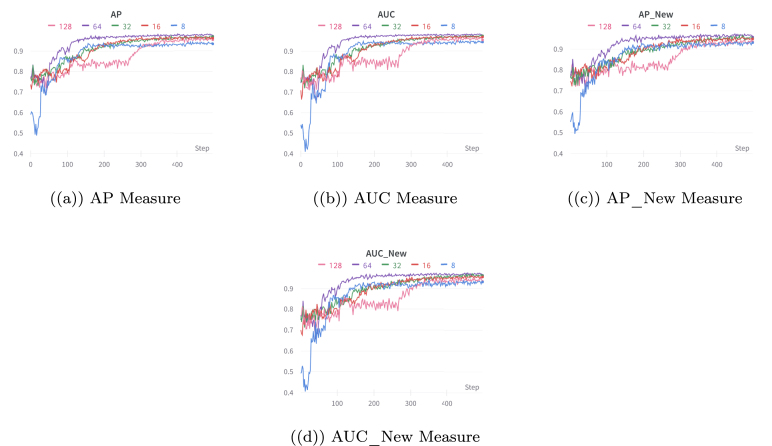
Illustration of the performance of VarGATDyn with different hidden dimensions for the link prediction and new link prediction tasks on the Enron dataset. The AP and AUC criteria are used to evaluate the models’ performance over 500 epochs. The graph shows that a hidden dimension of 64 achieved the best performance, as indicated by the purple colour in the graph.

### 4.4 Settings

We use Glorot initialisation [48] to set the model’s initial weights, and a learning rate of 0.01. The model is trained for 1000 epochs using the Adam optimiser [49]. To prevent overfitting, we apply early stopping on a validation set and stop the training process if the validation accuracy does not improve for 10 consecutive stages. In addition, we conduct a series of experiments to optimise and tune the model’s parameters, including alpha, m, and the hidden representation dimensions (all in Section 4.6). The final hidden representation dimension remains constant at 64 across all datasets. For the alpha value, it is configured to 0.5 for Enron, 0.6 for Facebook, and 0.3 for all other datasets. As for the final value of m, it is designated as 4 for Enron, 3 for Facebook, and 2 for the remaining datasets.

**Table 5:** Impact of K on the model’s performance in terms of AUCs and AP across the two dynamic link prediction tasks.

	Metric	K=1	K=2	K=3	K=4	K=5
Enron	AUC of Link prediction	92.75 ± 0.6	97.48 ± 0.7	97.88 ± 0.8	**98.71 ± 0.8**	98.07 ± 0.7
	AP of Link prediction	93.46 ± 0.6	97.64 ± 0.6	97.92 ± 0.7	**98.61 ± 0.7**	98.08 ± 0.8
	AUC of new Link prediction	86.83 ± 0.5	96.69 ± 0.6	96.96 ± 0.6	**98.09 ± 0.6**	97.55 ± 0.5
	AP of new Link prediction	88.91 ± 0.5	96.89 ± 0.6	96.69 ± 0.6	**97.77 ± 0.7**	96.99 ± 0.6
	Mean of AUCs	89.79 ± 0.6	97.08 ± 0.7	97.42 ± 0.7	**98.40 ± 0.7**	97.81 ± 0.6
	Mean of APs	91.18 ± 0.6	97.26 ± 0.6	97.30 ± 0.7	**98.19 ± 0.7**	97.53 ± 0.7

Colab	AUC of Link prediction	91.44 ± 0.6	97.67 ± 0.6	**97.80 ± 0.8**	97.19 ± 0.7	97.64 ± 0.6
	AP of Link prediction	92.91 ± 0.6	97.73 ± 0.6	97.78 ± 0.7	97.43 ± 0.6	**97.90 ± 0.8**
	AUC of new Link prediction	84.56 ± 0.5	96.72 ± 0.6	**97.91 ± 0.6**	96.32 ± 0.6	96.60 ± 0.6
	AP of new Link prediction	85.59 ± 0.5	97.20 ± 0.7	**97.20 ± 0.7**	96.39 ± 0.6	95.70 ± 0.7
	Mean of AUCs	88.00 ± 0.6	97.19 ± 0.6	**97.85 ± 0.7**	96.75 ± 0.7	97.12 ± 0.6
	Mean of APs	89.25 ± 0.6	96.46 ± 0.7	**97.49 ± 0.7**	96.91 ± 0.6	96.80 ± 0.8

Facebook	AUC of Link prediction	92.42 ± 0.4	96.71 ± 0.6	**96.89 ± 0.7**	96.50 ± 0.7	96.09 ± 0.6
	AP of Link prediction	91.84 ± 0.6	95.94 ± 0.6	**96.12 ± 0.7**	95.62 ± 0.7	95.33 ± 0.7
	AUC of new Link prediction	89.86 ± 0.4	96.10 ± 0.7	**96.78 ± 0.7**	96.11 ± 0.6	95.89 ± 0.6
	AP of new Link prediction	88.22 ± 0.5	95.01 ± 0.7	**95.56 ± 0.7**	95.09 ± 0.7	94.77 ± 0.8
	Mean of AUCs	91.14 ± 0.4	96.40 ± 0.7	**96.83 ± 0.7**	96.30 ± 0.7	95.99 ± 0.6
	Mean of APs	90.03 ± 0.6	95.47 ± 0.7	**95.84 ± 0.7**	95.35 ± 0.7	95.05 ± 0.8

### 4.5 Results analysis

**Dynamic Link Prediction**: In Table 2, we present the results of the various baselines against the proposed VarGATDyn in the dynamic link prediction task, reported in terms of AUC and AP, respectively. We note that VarGATDyn significantly outperforms all the baselines across the five datasets. In the Enron dataset, VarGATDyn exhibits a 4.17% improvement in terms of AP and a 4.54% improvement in AUC compared to the best previous result. In the Colab dataset, VarGATDyn significantly outperforms all of the baselines, with improvements of 5.87% in terms of AP and 6.58% in AUC, followed by HTGN and AMCNet, respectively. A similar trend is observed across the Facebook, UCI and Cora datasets, where VarGATDyn achieves the highest performance in terms of AP and AUC, followed by SI-VGRNN which consistently achieves the second-highest performance. Upon further examination, we compared our method with SI-VGRNN, VGRNN, and DySAT, widely recognised as top-performing techniques, using two larger-scale datasets, LFB and Yelp. The results, showcased in Table 4, unequivocally reveal our method’s superiority in two link prediction tasks, boasting higher AUC and AP values compared to the others.

**Dynamic New Link Prediction**:

Similarly, based on Table 3, our proposed model, VarGATDyn, demonstrated significant performance gains across the five datasets. Specifically, VarGATDyn achieved AP scores that were notably higher than the next best models: a 5.65% point improvement over AMCNet in the Enron dataset, 9.20% points in Colab, 8.84% points in Facebook, 3.96% points in UCI, and 3.66% points in Cora. These results underscore the superior performance of VarGATDyn in handling dynamic graph scenarios, clearly reflected in the higher AUC and AP values compared to alternative approaches.

**Fig. 5:**
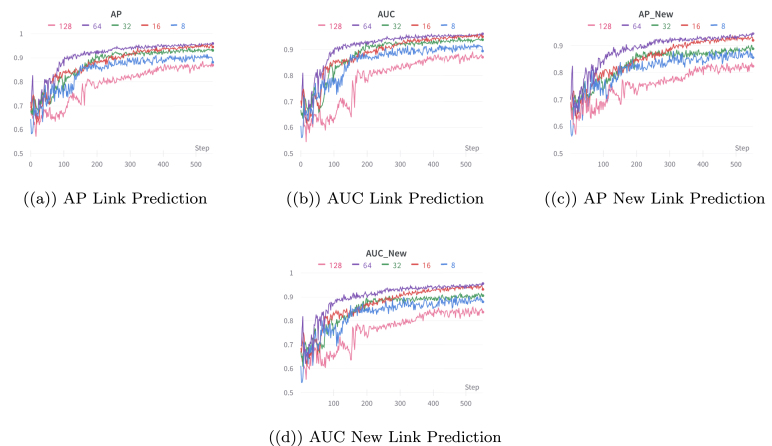
Comparison of VarGATDyn’s performance on the Colab dataset for link prediction and new link prediction tasks with various hidden dimensions. The model’s performance is evaluated using AP and AUC criteria over 500 epochs. The results show that using a hidden dimension of 64 yields the best performance, as depicted by the purple colour in the graph.

**Fig. 6:**
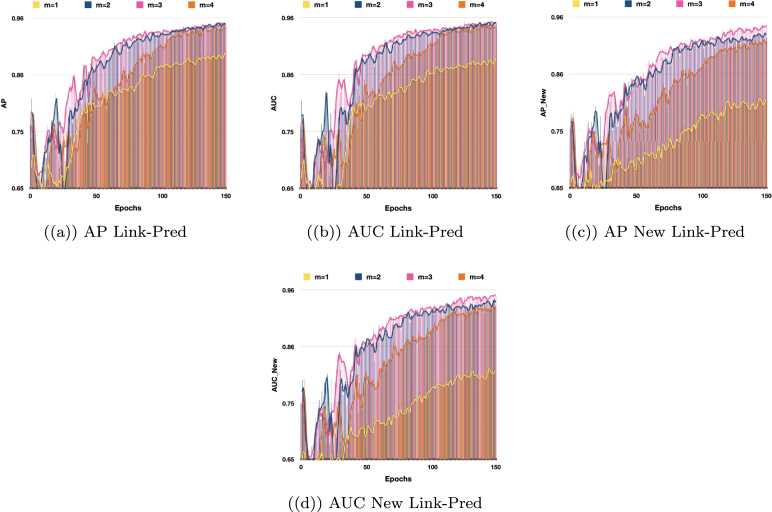
Performance comparison of VarGATDyn with different values of m for link prediction and new link prediction tasks on the Colab dataset. AP and AUC criteria are used to evaluate the models’ performance over 150 epochs. The chart shows the impact of different values of m on the model’s performance, with the best performance achieved at m=3 (represented by the colour pink).

**Fig. 7:**
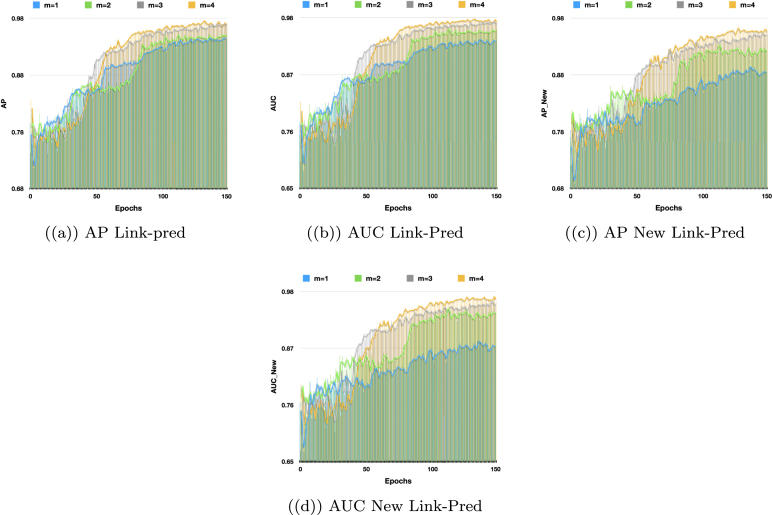
The performance of VarGATDyn on link prediction and new link prediction tasks using various values of m on the Enron dataset is compared in this study. The models were evaluated based on AP and AUC criteria over a period of 150 epochs, and the chart illustrates the effect of different m values on the model’s performance. The results indicate the model achieved the best performance with m=4 (represented by the yellow colour).

### 4.6 Impact of hyperparameters

To further study the impact of parameters used to train VarGATDyn, we conduct a series of experiments to observe the impact of K (Table 5), the number of mixtures used in GMM-VGAEs, on performing the model in both prediction tasks. The experiments are carried out on three datasets, namely, Enron, Colab, and Facebook. We observe that the highest performance gain was achieved when increasing K from 1 to 2, however, the performance slightly changed when increasing K from 2 to 5. This trend was observed across all datasets for both prediction tasks. We also examined the impact of different values of alpha on the performance of VarGATDyn in both prediction tasks. To determine the optimal value for alpha for each dataset, we uniformly sampled the range of [0, 1] and selected the value that produced the best average result across the four criteria (i.e., AUC and AP for link prediction, as well as AUC and AP for the new link prediction tasks). The results presented in Fig. 2 demonstrate that the worst performance is obtained when alpha is set to zero.

Additionally, we conducted a study to examine how the hidden dimensions affect the performance of the VarGATDyn model. The performance of the models has evaluated over 500 epochs (instead of 1000 epochs, for clarity) for the Enron and Colab datasets, as shown in Fig. 4, Fig. 5, respectively. Our findings reveal that the hidden dimension of size 64 consistently outperforms other dimensions in both metrics for the dynamic link prediction and dynamic new link prediction tasks across both datasets.

### 4.7 Computational complexity analysis

The VarGATDyn model incorporates a sophisticated integration of GMM with VGAE and GAT, aimed at enhancing dynamic graph representation learning. The primary computational load stems from the model’s GCN-based encoder, which is predominantly influenced by the number of edges, leading to a complexity of O(|E|H). Here H represents the hidden dimension size and |E| is the count of edges. Additionally, the inclusion of GAT introduces an augmented complexity of $O\left(\left|E\right|H^{2}\right)$, reflecting the computation of attention coefficients for each edge, scaled by the square of the feature dimension. Further complexity arises from the iterative learning process, which repeats these computations m times across T timesteps, culminating in an overall computational demand of $O\left(mT\left|E\right|H^{2}\right)$. This comprehensive approach underpins the model’s capability to effectively capture and integrate complex structural and temporal dynamics within evolving graph structures. We also introduced Table 6 , which shows the complexity of other models.

**Fig. 8:**
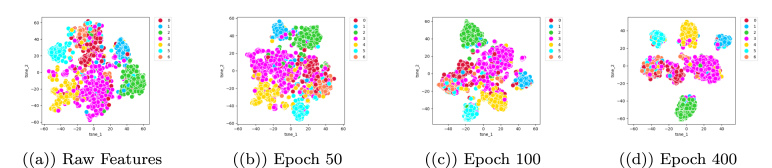
Visualisation of the embeddings learnt by VarGATDyn over time on the Cora dataset. Each colour represents a cluster, with (a) showing the embeddings at the beginning of training where clusters are not distinguishable, (b) at epoch number 50 where clusters are more clear with a silhouette score of 16.70, (c) at epoch 100 where clusters become even more distinct with a silhouette score of 29.98, and (d) at epoch 400 where clusters are almost completely separated with a silhouette score of 46.61.

**Table 6:** Time Complexity of different methods.

Method	Time complexity
DynAE	O(T|E|+|V|)
DynRNN	$O\left(T\left|V\right|H^{2}\right)$
DynAERNN	$O\left(T\left|V\right|H^{2}+T\left(\left|E\right|+\left|V\right|\right)\right)$
HTGN	$O\left(T\left|V\right|H^{2}+\left|E\right|H^{2}\right)$
DySAT	$O\left(T\left|V\right|H^{2}+\left|E\right|H^{2}\right)$
VGRNN	$O\left(T\left|V\right|H^{2}\right)+O\left(\left|E\right|\right)$
VarGATDyn	$O\left(mT\left|E\right|H^{2}\right)$

### 4.8 Ablation study

To highlight the significance of the GMM in enhancing the performance of the VarGATDyn model, we conducted an ablation study in which we removed the GMM component from the VarGATDyn model. The outcomes, as shown in Fig. 3, revealed a considerable improvement in the results for the Enron, Colab, and Facebook datasets, with a boost observed across all metrics and in both link prediction tasks. This enhancement indicates the crucial role played by the GMM in achieving the performance gains demonstrated by the VarGATDyn model. Moreover, we explore the multiple-learning technique and its impact on the performance of VarGATDyn. Our investigation shows that the model’s performance can be significantly improved by utilising this technique. To conduct the investigation, we first applied our model without using the multiple-learning technique and then incorporated it by repeating the process multiple times to determine the optimal outcome. The results of our investigation on the Colab dataset are displayed in Fig. 6 and on the Enron dataset in Fig. 7. As depicted in Fig. 6, Fig. 7, when m=1 and the multiple-learning technique is not applied, the results are significantly lower than when m=2 and the technique is utilised. The optimal value of m varies across different datasets. For instance, in the Enron dataset, the best performance was achieved with m=4, indicating that the model benefited from a higher number of learning iterations. In contrast, the Colab dataset achieved the best performance with m=2, suggesting that just one extra iteration was sufficient to improve the performance.

### 4.9 Qualitative analysis

In our study, we evaluated the effectiveness of our learned embeddings over time using the Cora dataset. Specifically, we employed a clustering task and the silhouette metric to assess how well the clusters of learned embeddings were modelled over time. To visualise our results, we plotted the learned embeddings in a two-dimensional space using t-SNE, as shown in Fig. 8. Our results indicate that the learned embeddings were effectively encoded, as demonstrated by the clear separation between clusters, which became increasingly distinct over time. This suggests that our model was able to effectively capture the underlying structure and relationships within the data, enabling accurate clustering and classification.

## 5 Conclusion and future work

We proposed VarGATDyn, an integrated variational model designed for learning node representations in dynamic graphs. VarGATDyn uses an attention mechanism with Markovian assumptions for temporal modelling, and a GMM within the variational framework to infer the multimodal nature of the data. Additionally, VarGATDyn learned through an iterative process involving inference, generation, and learning at each time step, referred to as a multiple-learning technique, resulting in a robust and comprehensive learning process. Our experimental results have clearly demonstrated that VarGATDyn has achieved state-of-the-art performance, surpassing existing methods in dynamic and new link prediction tasks. This showcases the effectiveness of VarGATDyn in learning dynamic graph representations. Moreover, in our proposed method, VGAEs are chiefly centred on the reconstruction of the adjacency matrix, sidelining the features matrix. A promising direction for future investigations involves incorporating both the feature and adjacency matrices during the reconstruction phase. We hypothesise that such integration might enhance accuracy. Although our model’s performance has been validated across a spectrum of datasets and challenges, we aim to further scrutinise its capabilities on more expansive and heterogeneous datasets. This will provide deeper insights into its versatility and efficacy in diverse research contexts and applications moving forward.

## CRediT authorship contribution statement

**Soheila Molaei:** Writing – review & editing, Writing – original draft, Visualization, Project administration, Methodology, Investigation, Data curation, Conceptualization. **Ghazaleh Niknam:** Writing – original draft, Methodology, Investigation, Data curation, Conceptualization. **Ghadeer O. Ghosheh:** Writing – original draft, Methodology, Investigation, Data curation, Conceptualization. **Vinod Kumar Chauhan:** Writing – original draft, Investigation, Data curation, Conceptualization. **Hadi Zare:** Writing – review & editing, Validation, Supervision, Methodology, Data curation, Conceptualization. **Tingting Zhu:** Writing – review & editing, Validation, Methodology, Data curation, Conceptualization. **Shirui Pan:** Writing – review & editing, Validation, Supervision, Methodology, Investigation, Conceptualization. **David A. Clifton:** Writing – review & editing, Supervision, Methodology, Funding acquisition, Data curation, Conceptualization.

## Declaration of competing interest

The authors declare that they have no known competing financial interests or personal relationships that could have appeared to influence the work reported in this paper.

## Data Availability

Data will be made available on request.

## References

[1] A. Fout, J. Byrd, B. Shariat, A. Ben-Hur, Protein interface prediction using graph convolutional networks, in: Proceedings of the 31st International Conference on Neural Information Processing Systems, 2017, pp. 6533–6542.

[2] Y. Liu, X. Shi, L. Pierce, X. Ren, Characterizing and forecasting user engagement with in-app action graph: A case study of snapchat, in: Proceedings of the 25th ACM SIGKDD International Conference on Knowledge Discovery & Data Mining, 2019, pp. 2023–2031.

[3] Zhao L., Song Y., Zhang C., Liu Y., Wang P., Lin T., Deng M., Li H. (2019). T-GCN: A temporal graph convolutional network for traffic prediction. IEEE Trans. Intell. Transp. Syst..

[4] Huang Z., Tang Y., Chen Y. (2022). A graph neural network-based node classification model on class-imbalanced graph data. Knowl.-Based Syst..

[5] Agarwal C., Lakkaraju H., Zitnik M. (2021). Uncertainty in Artificial Intelligence.

[6] Molaei S., Bousejin N.G., Zare H., Jalili M., Pan S. (2021). Learning graph representations with maximal cliques. IEEE Trans. Neural Netw. Learn. Syst..

[7] He C., Cheng J., Fei X., Weng Y., Zheng Y., Tang Y. (2023). Community preserving adaptive graph convolutional networks for link prediction in attributed networks. Knowl.-Based Syst..

[8] Mudiyanselage T.B., Lei X., Senanayake N., Zhang Y., Pan Y. (2022). Predicting CircRNA disease associations using novel node classification and link prediction models on graph convolutional networks. Methods.

[9] Niknam G., Molaei S., Zare H., Clifton D., Pan S. (2023). Graph representation learning based on deep generative gaussian mixture models. Neurocomputing.

[10] Molaei S., Bousejin N.G., Zare H., Jalili M. (2021). Deep node clustering based on mutual information maximization. Neurocomputing.

[11] Xu M., Wang H., Ni B., Guo H., Tang J. (2021). International Conference on Machine Learning.

[12] Yang H., Wang J., Duan R., Yan C. (2023). DCOM-GNN: A deep clustering optimization method for graph neural networks. Knowl.-Based Syst..

[13] L. Zhou, Y. Yang, X. Ren, F. Wu, Y. Zhuang, Dynamic network embedding by modeling triadic closure process, in: Proceedings of the Thirty-Second AAAI Conference on Artificial Intelligence and Thirtieth Innovative Applications of Artificial Intelligence Conference and Eighth AAAI Symposium on Educational Advances in Artificial Intelligence, 2018, pp. 571–578.

[14] Goyal P., Kamra N., He X., Liu Y. (2018). http://arxiv.org/abs/1805.11273.

[15] Shen F., Wang J., Zhang Z., Wang X., Li Y., Geng Z., Pan B., Lu Z., Zhao W., Zhu W. (2023). Long-term multivariate time series forecasting in data centers based on multi-factor separation evolutionary spatial–temporal graph neural networks. Knowl.-Based Syst..

[16] Manessi F., Rozza A., Manzo M. (2020). Dynamic graph convolutional networks. Pattern Recognit..

[17] Chen J., Zhang J., Xu X., Fu C., Zhang D., Zhang Q., Xuan Q. (2019). E-lstm-d: A deep learning framework for dynamic network link prediction. IEEE Trans. Syst. Man Cybern. Syst..

[18] J. Li, Z. Han, H. Cheng, J. Su, P. Wang, J. Zhang, L. Pan, Predicting path failure in time-evolving graphs, in: Proceedings of the 25th ACM SIGKDD International Conference on Knowledge Discovery & Data Mining, 2019, pp. 1279–1289.

[19] Seo Y., Defferrard M., Vandergheynst P., Bresson X. (2018). International Conference on Neural Information Processing.

[20] Narayan A., Roe P.H. (2018). Learning graph dynamics using deep neural networks. IFAC-PapersOnLine.

[21] A. Sankar, Y. Wu, L. Gou, W. Zhang, H. Yang, Dysat: Deep neural representation learning on dynamic graphs via self-attention networks, in: Proceedings of the 13th International Conference on Web Search and Data Mining, 2020, pp. 519–527.

[22] Rezende D.J., Mohamed S., Wierstra D. (2014). International Conference on Machine Learning.

[23] E. Hajiramezanali, A. Hasanzadeh, N. Duffield, K. Narayanan, M. Zhou, X. Qian, Variational graph recurrent neural networks, in: Proceedings of the 33rd International Conference on Neural Information Processing Systems, 2019, pp. 10701–10711.

[24] Aneja J., Schwing A., Kautz J., Vahdat A. (2021). A contrastive learning approach for training variational autoencoder priors. Adv. Neural Inf. Process. Syst..

[25] Kostantinos N. (2000). Advanced Signal Processing Handbook: Theory and Implementation for Radar, Sonar, and Medical Imaging Real Time Systems.

[26] Goodfellow I., Bengio Y., Courville A. (2016).

[27] Mahdavi S., Khoshraftar S., An A. (2019). Joint European Conference on Machine Learning and Knowledge Discovery in Databases.

[28] Skarding J., Gabrys B., Musial K. (2021). Foundations and modeling of dynamic networks using dynamic graph neural networks: A survey. IEEE Access.

[29] M. Defferrard, X. Bresson, P. Vandergheynst, Convolutional neural networks on graphs with fast localized spectral filtering, in: Proceedings of the 30th International Conference on Neural Information Processing Systems, 2016, pp. 3844–3852.

[30] Gers F.A., Schraudolph N.N., Schmidhuber J. (2002). Learning precise timing with LSTM recurrent networks. J. Mach. Learn. Res..

[31] A. Pareja, G. Domeniconi, J. Chen, T. Ma, T. Suzumura, H. Kanezashi, T. Kaler, T. Schardl, C. Leiserson, Evolvegcn: Evolving graph convolutional networks for dynamic graphs, in: Proceedings of the AAAI Conference on Artificial Intelligence, Vol. 34, No. 04, 2020, pp. 5363–5370.

[32] Chen J., Wang X., Xu X. (2022). GC-LSTM: Graph convolution embedded LSTM for dynamic network link prediction. Appl. Intell..

[33] S. Pan, R. Hu, G. Long, J. Jiang, L. Yao, C. Zhang, Adversarially regularized graph autoencoder for graph embedding, in: Proceedings of the 27th International Joint Conference on Artificial Intelligence, 2018, pp. 2609–2615.

[34] Charte D., Charte F., del Jesus M.J., Herrera F. (2020). An analysis on the use of autoencoders for representation learning: Fundamentals, learning task case studies, explainability and challenges. Neurocomputing.

[35] Lei K., Qin M., Bai B., Zhang G., Yang M. (2019). IEEE INFOCOM 2019-IEEE Conference on Computer Communications.

[36] B. Hui, P. Zhu, Q. Hu, Collaborative graph convolutional networks: Unsupervised learning meets semi-supervised learning, in: Proceedings of the AAAI Conference on Artificial Intelligence, Vol. 34, No. 04, 2020, pp. 4215–4222.

[37] Priebe C.E., Conroy J.M., Marchette D.J., Park Y. (2005). Scan statistics on enron graphs. Comput. Math. Organization Theory.

[38] Xu K.S., Hero A.O. (2014). Dynamic stochastic blockmodels for time-evolving social networks. IEEE J. Sel. Top. Sign. Proces..

[39] Rahman M., Al Hasan M. (2016). Joint European Conference on Machine Learning and Knowledge Discovery in Databases.

[40] B. Viswanath, A. Mislove, M. Cha, K.P. Gummadi, On the evolution of user interaction in facebook, in: Proceedings of the 2nd ACM Workshop on Online Social Networks, 2009, pp. 37–42.

[41] Fathy A., Li K. (2020). Advances in Knowledge Discovery and Data Mining: 24th Pacific-Asia Conference, PAKDD 2020, Singapore, May 11–14, 2020, Proceedings, Part I 24.

[42] Sen P., Namata G., Bilgic M., Getoor L., Galligher B., Eliassi-Rad T. (2008). Collective classification in network data. AI Mag..

[43] X. Liu, P.-C. Hsieh, N. Duffield, R. Chen, M. Xie, X. Wen, Real-time streaming graph embedding through local actions, in: Companion Proceedings of the 2019 World Wide Web Conference, 2019, pp. 285–293.

[44] Goyal P., Chhetri S.R., Canedo A. (2020). dyngraph2vec: Capturing network dynamics using dynamic graph representation learning. Knowl.-Based Syst..

[45] M. Yang, M. Zhou, M. Kalander, Z. Huang, I. King, Discrete-time Temporal Network Embedding via Implicit Hierarchical Learning in Hyperbolic Space, in: Proceedings of the 27th ACM SIGKDD Conference on Knowledge Discovery & Data Mining, 2021, pp. 1975–1985.

[46] G. Zhang, T. Ye, D. Jin, Y. Li, An attentional multi-scale co-evolving model for dynamic link prediction, in: Proceedings of the ACM Web Conference 2023, 2023, pp. 429–437.

[47] T.N. Kipf, M. Welling, Variational Graph Auto-Encoders, in: NIPS Workshop on Bayesian Deep Learning, 2016, pp. 1–12.

[48] X. Glorot, Y. Bengio, Understanding the difficulty of training deep feedforward neural networks, in: Proceedings of the Thirteenth International Conference on Artificial Intelligence and Statistics, 2010, pp. 249–256.

[49] D.P. Kingma, J. Ba, Adam: A Method for Stochastic Optimization, in: ICLR (Poster), 2015, pp. 1–15.

